# Ductal Carcinoma In Situ of the Breast: An Analysis of 12 Years of Experience From a Tertiary Center

**DOI:** 10.7759/cureus.89987

**Published:** 2025-08-13

**Authors:** Hüseyin Tepetam, Mustafa Mert Hanilce, Cemal Ugur Dursun, Duygu Gedik, Sule Karabulut Gul

**Affiliations:** 1 Radiation Oncology, Kartal Dr. Lütfi Kırdar City Hospital, Istanbul, TUR

**Keywords:** adjuvant radiotherapy, breast cancer, breast-conserving surgery, ductal carcinoma in situ, high-grade dcis

## Abstract

Background

Ductal carcinoma in situ (DCIS) is a non-invasive breast malignancy that accounts for approximately one-fifth of new breast cancer diagnoses in developed countries. While not life-threatening, DCIS is a potential precursor to invasive carcinoma, and optimal management remains a matter of debate. Adjuvant radiotherapy after breast-conserving surgery reduces the risk of local recurrence, and hypofractionated regimens have emerged as an effective and convenient alternative to conventional fractionation. This study aimed to compare acute and late toxicity profiles between hypofractionated and conventional radiotherapy in DCIS patients, with a particular focus on the impact of boost administration.

Methodology

This retrospective study included 97 female patients with histologically confirmed DCIS treated between January 2013 and January 2025. Patients with invasive components, prior malignancy, or incomplete data were excluded. Radiotherapy was delivered using three-dimensional conformal radiotherapy (n = 43) or intensity-modulated radiotherapy (n = 54), with conventional fractionation (50 Gy/25 fractions, used in 30.9% (30 patients)) or hypofractionated regimens (40-42.6 Gy/15-16 fractions, used in 69.1% (67 patients)). A sequential boost of 10 Gy was applied in high-risk cases. Data were analyzed using SPSS version 27.0 (IBM Corp., Armonk, NY, USA), with significance set at p-values <0.05.

Results

A total of 97 DCIS patients received adjuvant radiotherapy (mean age: 51 years). Hypofractionated radiotherapy was used in 69.1% (67 patients), and conventional fractionation in 30.9% (30 patients). Acute toxicity occurred in 60.8% (59 patients), while late toxicity was rare (8.2% (8 patients)) and similar across groups. Boost administration was associated with increased acute toxicity in the hypofractionated group (p = 0.044) but not in the conventional group. Only one local recurrence was observed during follow-up.

Conclusions

Our findings confirm that both hypofractionated and conventional radiotherapy provide excellent local control and acceptable toxicity in DCIS patients, with boost administration significantly increasing acute toxicity in hypofractionated regimens. Despite these differences, long-term outcomes remain favorable, supporting the role of individualized risk-adapted radiotherapy strategies in DCIS management.

## Introduction

Ductal carcinoma in situ (DCIS) is a non-invasive breast malignancy characterized by the proliferation of neoplastic epithelial cells confined within the ductal-lobular system, without evidence of basement membrane invasion [1]. With the widespread adoption of screening mammography, the incidence of DCIS has markedly increased over the past three decades, now accounting for approximately 20-25% of all newly diagnosed breast cancers in developed countries [2]. Although DCIS itself is not life-threatening, it is considered a potential precursor to invasive ductal carcinoma, and its management remains a subject of ongoing clinical debate [2,3].

DCIS represents a biologically heterogeneous disease, ranging from indolent lesions with minimal malignant potential to high-grade forms with a significant risk of progression [3,4]. This variability presents a challenge in selecting appropriate treatment strategies, particularly in light of increasing concerns about overtreatment in patients with low-risk features [1-4]. Standard management options include breast-conserving surgery (BCS) or mastectomy, often followed by adjuvant radiotherapy and endocrine therapy in hormone receptor-positive cases [5]. These interventions aim to reduce the risk of ipsilateral breast tumor recurrence, especially invasive recurrence, which is associated with increased breast cancer-specific mortality [6].

In parallel with discussions on patient selection for adjuvant radiotherapy, attention has also shifted toward optimizing radiation delivery methods. Hypofractionated radiotherapy (HFRT), involving larger doses per fraction over a shorter overall treatment time, has gained prominence due to its convenience, comparable efficacy, and favorable toxicity profile [2,3,7]. As a result, hypofractionation is increasingly being adopted in early-stage breast cancer management, including selected DCIS cases, reflecting a broader move toward personalized and resource-efficient care [3,4,8].

Retrospective analyses from single institutions provide meaningful contributions to our understanding of DCIS management in real-world clinical settings. Although limited by small sample sizes or low event rates, such studies can offer valuable insights into treatment approaches, recurrence patterns, and potential clinicopathological associations. In this study, we present a 12-year retrospective analysis of DCIS cases treated at a high-volume tertiary care center. The primary objective was to compare the acute and late toxicity profiles of hypofractionated versus conventional fractionation radiotherapy regimens, incorporating the impact of boost administration. This explicit statement of aims reflects the study’s focus on toxicity outcomes, while acknowledging that recurrence data play a supportive role due to the rarity of events in our cohort.

## Materials and methods

Study design and patient selection

**Figure 1:**
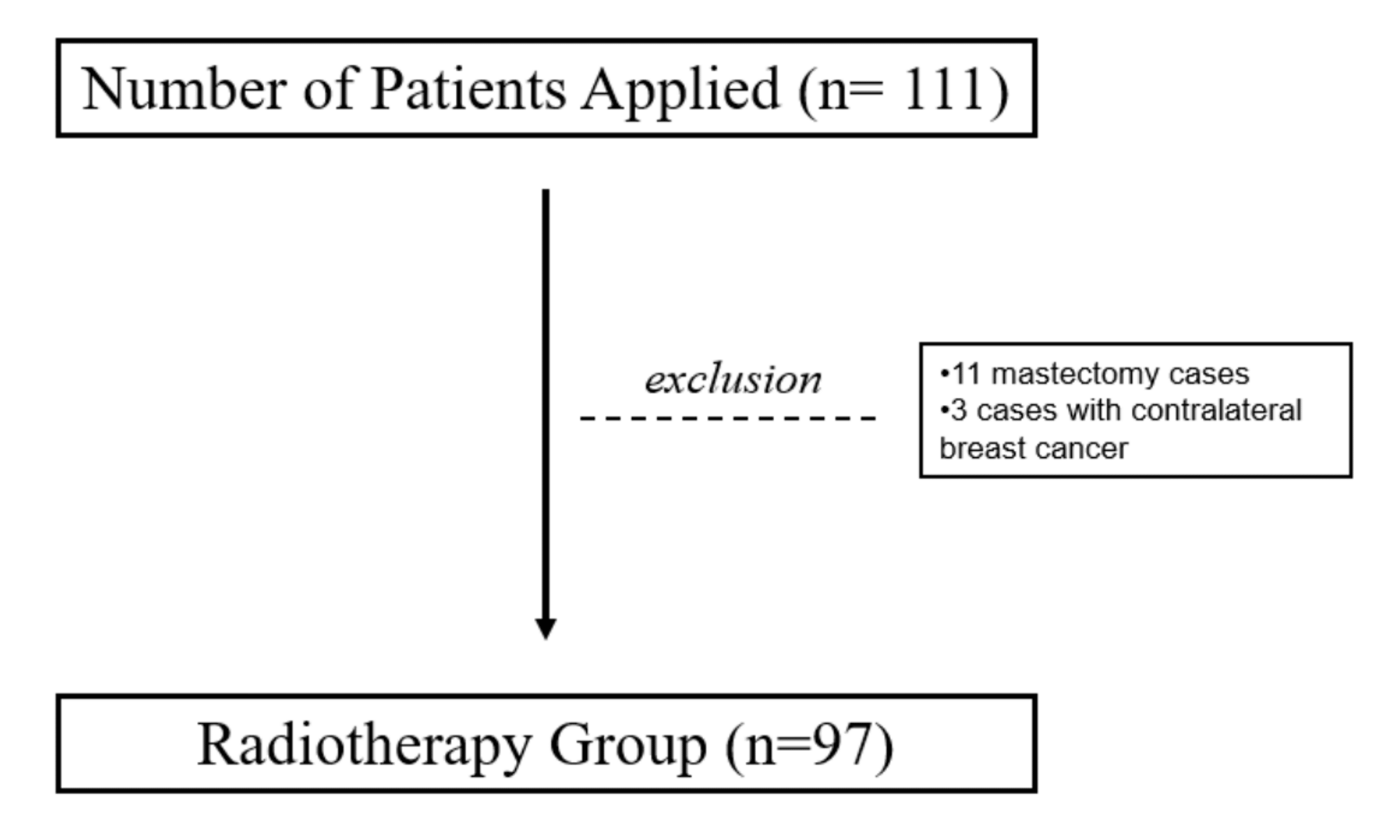
Flowchart of patient selection (excluding 11 mastectomy cases and three cases with synchronous contralateral breast cancer).

This retrospective cohort study was conducted at the Department of Radiation Oncology, Kartal Dr. Lutfi Kirdar City Hospital, Istanbul, Türkiye. Female patients with a postoperative histopathological diagnosis of DCIS who received radiotherapy between January 2013 and January 2025 were included. A total of 111 patients were initially screened. Inclusion criteria included planned or administered radiotherapy at our institution and the availability of complete clinical and pathological records. Exclusion criteria were male sex, age under 18 years, previous malignancy or radiotherapy, lack of clinical indication for radiotherapy, and presence of an invasive component on final pathology. Based on these criteria, 97 patients were included in the final analysis (Figure 1). Ethical approval was obtained from the Institutional Review Board (approval number: 2025/010.99/15/24).

Data collection and variables

Clinical, pathological, and treatment data were collected from the hospital’s electronic health records and the departmental database. Data were independently extracted by two radiation oncologists, with discrepancies resolved through consensus. Collected variables included age at diagnosis, family history of breast cancer, tumor size, nuclear grade, comedonecrosis, estrogen receptor (ER), progesterone receptor (PR), and human epidermal growth factor receptor 2 (HER2) status. Treatment-related data included type of surgery, margin width, axillary staging, endocrine therapy, radiotherapy modality (three-dimensional conformal radiotherapy (3D-CRT) or intensity-modulated radiotherapy (IMRT)), fractionation regimen, and boost administration. Toxicity outcomes were assessed using physician-recorded evaluations during follow-up.

Radiotherapy simulation and treatment protocol

All patients underwent CT-based simulation in the supine position with both arms elevated. Immobilization was achieved with a standard breast board or a customized alpha cradle. CT images were acquired with a 2 mm slice thickness and transferred to the treatment planning system for target volume delineation and dose calculation. Radiotherapy was delivered using either 3D-CRT (n = 43) or IMRT (n = 54). Fractionation regimens included conventional fractionation (50 Gy in 25 fractions) and hypofractionation (40-42.6 Gy in 15-16 fractions). A sequential photon boost of 10 Gy in four to five fractions was prescribed for patients at high risk of local recurrence, as defined by our institutional criteria: age under 40 years, presence of comedonecrosis, high histological grade, and positive or close surgical margins. Boost indication was based on our predefined clinical protocol. In total, 63 patients received photon boost irradiation. The boost volume was delineated using a combination of preoperative imaging (mammography, ultrasound, and MRI when available) and radiology reports, postoperative ultrasound evaluation, and the presence of surgical clips or markers placed during tumor excision to define the tumor bed. The clinical target volume for boost encompassed the surgical cavity with an appropriate margin, adjusted for anatomical boundaries and normal tissue constraints. All treatment planning and delivery were performed according to our institution’s accepted dose constraints and planning objectives. Patients with ER-positive disease were offered adjuvant hormonal therapy in accordance with institutional guidelines.

Follow-up and toxicity evaluation

Patients were assessed weekly during the course of radiotherapy for the monitoring and documentation of acute treatment-related side effects. Upon completion of radiotherapy, the first follow-up visit was scheduled at six weeks, followed by a second visit at three months. Subsequently, patients were monitored every three months for the first two years, every five months until the fifth year, and annually thereafter.

Patients were assessed weekly during radiotherapy for acute toxicity. After treatment, follow-up was scheduled at six weeks, three months, every three months for two years, every five months until year five, and annually thereafter. Acute toxicities were defined as those occurring during or within three months of radiotherapy, while late toxicities were defined as those occurring afterward. Toxicities were graded using Common Terminology Criteria for Adverse Events version 4.0, with acute toxicities including dermatitis, breast edema, pain, and pruritus, and late toxicities including fibrosis, telangiectasia, breast edema, pain, and skin pigmentation changes [9]. All toxicity assessments were performed by physicians from our department. Subjective toxicities were evaluated based on patient-reported outcomes, and in cases where patient reporting was unavailable, these toxicities were systematically inquired about and documented by the treating physician.

Statistical analysis

All analyses were performed using SPSS version 27.0 (IBM Corp., Armonk, NY, USA). Descriptive statistics (means, medians, standard deviations, frequencies) were used to summarize data. Comparisons of categorical variables were made using chi-square or Fisher’s exact tests. A p-value <0.05 was considered significant. No adjustments for multiple comparisons were made.

## Results

Between January 2013 and January 2025, 111 patients were diagnosed with postoperative DCIS at our institution. Of these, 97 patients who received adjuvant radiotherapy were included in the final analysis. The median follow-up was 36 months (range = 2-144 months), and the mean age at diagnosis was 51 years (range = 31-77 years). A family history of breast cancer in first- or second-degree relatives was reported in 23 (23.7%) patients. All patients underwent BCS, with 38 (39.2%) patients requiring re-excision for margin clearance, achieving final surgical margins of ≥2 mm in 76.3% of cases, increasing to 81% after 2016. Axillary staging was performed in 55 (56.8%) patients, while the 11 patients not receiving radiotherapy had undergone mastectomy and were excluded from the analysis.

High-grade lesions were present in 40.2% of tumors, intermediate-grade in 36.1%, and low-grade in 23.7%. The mean tumor diameter was 1.4 cm (range = 0.2-8.5 cm). ER positivity was detected in 79 (81.5%) patients, and HER2 positivity in 19 (19.5%) patients. Hormone receptor status was unavailable in 17 (17.5%) patients.

HFRT was administered in 69.1% of patients, using either 40 Gy in 15 (40.3%) fractions or 42.6 Gy in 16 (59.7%) fractions. Conventional radiotherapy (CRT; 50 Gy in 25 fractions) was delivered to 30.9% of patients. A radiotherapy boost was delivered to 48 patients in the HFRT group (71.6%) and 15 patients in the CRT group (50%). Among those receiving a boost, 33 (52.4%) patients received 10 Gy in 4 fractions, and 30 (47.6%) patients received 10 Gy in five fractions.

Acute toxicity occurred in 59 (60.8%) patients, most commonly as radiation dermatitis, including Grade 1 in 41 patients, Grade 2 in 11 patients, and Grade 3 in two patients. Late toxicity was observed in eight (8.2%) patients, all of which were Grade 1. When comparing HFRT and CRT, acute toxicity occurred in 41 (61.2%) patients versus 18 (60.0%) patients (p = 0.911), and late toxicity in 6 (9.0%) versus 2 (6.7%) patients (p = 0.705). Among patients not receiving a boost, acute toxicity was seen in eight (42.1%) HFRT patients and nine (60.0%) CRT patients (p = 0.300), while late toxicity occurred in two (10.5%) patients and 1 (6.7%) patient, respectively (p = 1.000). In the HFRT group, acute toxicity was significantly more common in boost recipients (68.8%) compared to non-recipients (42.1%) (p = 0.044), while late toxicity rates were similar (8.3% vs. 10.5%) (p = 0.777). In the CRT group, boost administration did not influence acute or late toxicity rates (acute = 60.0%, late = 6.7%; p = 1.000 for both). Overall, boost administration across all patients was associated with higher acute toxicity (66.7% with boost vs. 50.0% without boost) (p = 0.880), while late toxicity was comparable (7.9% vs. 8.8%) (p = 0.109). Comparing boost recipients between HFRT and CRT, acute toxicity was 68.8% versus 60.0% (p = 0.530), and late toxicity was 8.3% versus 6.7% (p = 0.832), with no significant differences observed.

Notably, only one patient treated with HFRT experienced a local recurrence, which was observed 60 months after treatment. No other locoregional or distant recurrences were observed. In conclusion, early toxicity was associated with boost administration in the HFRT group but not in the CRT group. No significant differences in overall toxicity profiles were observed between HFRT and CRT regimens, including in subgroup analyses. Detailed numerical outcomes are presented in Table 1.

**Table 1:** Comparison of acute and late toxicity rates by radiotherapy modality and boost administration. This table summarizes the comparison of acute and late toxicity outcomes according to radiotherapy modality (HFRT vs. CRT) and boost administration status. Statistical comparisons were performed using the chi-square test (a) or Fisher’s exact test (b), as appropriate. A p-value <0.05 was considered statistically significant. HFRT = hypofractionated radiotherapy; CRT = conventional radiotherapy; χ² = chi-square value

Group	Total patients (n)	Acute toxicity present, n (%)	Acute toxicity absent, n (%)	P-value (acute)	χ² (acute)	Late toxicity present, n (%)	Late toxicity absent, n (%)	P-value (late)	χ² (late)
HFRT	67	41 (61.2%)	26 (38.8%)	0.911^a^	0.012	6 (9.0%)	61 (91.0%)	0.705^b^	0.143
CRT	30	18 (60.0%)	12 (40.0%)			2 (6.7%)	28 (93.3%)		
HFRT with boost	48	33 (68.8%)	15 (31.3%)	0.530^a^	0.393	4 (8.3%)	44 (91.7%)	1.000^b^	-
CRT with boost	15	9 (60.0%)	6 (40.0%)			1 (6.7%)	14 (93.3%)		
HFRT without boost	19	8 (42.1%)	11 (57.9%)	0.300^a^	1.073	2 (10.5%)	17 (89.5%)	1.000^b^	-
CRT without boost	15	9 (60.0%)	6 (40.0%)			1 (6.7%)	14 (93.3%)		
HFRT with boost	48	33 (68.8%)	15 (31.3%)	0.044^a^	4.069	4 (8.3%)	44 (91.7%)	1.000^b^	-
HFRT without boost	19	8 (42.1%)	11 (57.9%)			2 (10.5%)	17 (89.5%)		
CRT with boost	15	9 (60.0%)	6 (40.0%)	1.000^a^	0	1 (6.7%)	14 (93.3%)	1.000^b^	-
CRT without boost	15	9 (60.0%)	6 (40.0%)			1 (6.7%)	14 (93.3%)		
Boost given	63	42 (66.7%)	21 (33.3%)	0.108^a^	2.574	5 (7.9%)	58 (92.1%)	1.000^b^	-
No boost	34	17 (50.0%)	17 (50.0%)			3 (8.8%)	31 (91.2%)		

## Discussion

Our findings demonstrate that both HFRT and CRT provide comparable local control and toxicity profiles in patients with DCIS, consistent with multiple studies that support the efficacy and safety of HFRT in this setting [10-12]. In particular, we observed that the use of a tumor bed boost in HFRT was associated with higher rates of acute toxicity, consistent with prior data from the BONBIS trial that demonstrated significantly increased rates of acute skin toxicities when a boost was administered [13-15]. We acknowledge that the median follow-up duration of 36 months is relatively short to definitively comment on long-term local control and recurrence outcomes; however, our findings are in line with existing literature demonstrating that adjuvant radiotherapy substantially improves local control in DCIS. The excellent overall local control in our cohort, with only one recurrence observed, aligns with long-term data showing that adjuvant radiotherapy substantially reduces local relapse rates in DCIS [16].

A major focus of our study was the impact of boost radiotherapy in DCIS management, an area of ongoing debate. The landmark TROG 07.01/BIG 3-07 randomized trial recently confirmed that boost radiotherapy significantly reduces local recurrence rates, with a five-year local failure rate of 3% compared to 7% without boost, reinforcing earlier cohort data demonstrating a hazard ratio (HR) of 0.68 for ipsilateral breast tumor recurrence and a protective effect in high-risk subgroups [17-19]. However, some studies suggest that this benefit may be modest or limited to specific patient groups, underscoring the need for individualized treatment plans [20].

Current clinical guidelines recommend considering boost administration in patients with high-risk features, including young age, high-grade DCIS, comedonecrosis, or close/positive surgical margins [19,21,22]. Our data, demonstrating higher rates of boost use in high-grade DCIS, align with these recommendations. Nevertheless, a meta-analysis indicated that the absolute local control benefit of boost may be more pronounced in positive margin scenarios and not significant for low-risk patients [10]. Emerging molecular assays could refine this further by identifying biologically high-risk DCIS phenotypes that would derive the most benefit from a boost [12,23].

The growing adoption of hypofractionated schedules has been driven by their convenience and comparable outcomes, including similar local control and acceptable toxicity profiles [24-26]. Our data align with these observations, demonstrating that HFRT with a boost achieved excellent local control while maintaining low late toxicity rates. However, we noted an increased incidence of acute skin toxicity when a boost was administered, a finding consistent with previous reports from phase II studies [27,28]. Notably, advanced treatment planning techniques such as simultaneous integrated boost (SIB) with IMRT have shown promise in minimizing dose to normal tissues while maintaining robust target coverage, supporting their role in optimizing cosmetic and oncologic outcomes [29]. Moreover, patient-reported data from recent studies emphasize that while HFRT with a boost may lead to slightly inferior cosmetic results compared to HFRT alone, these differences are generally minor and do not significantly impact overall quality of life [30].

As with any retrospective single-center analysis, our study is subject to inherent limitations, including potential selection bias and lack of randomization, as seen in similar institutional experiences [10,19]. A major limitation of our study is the median follow-up duration of 36 months, which is insufficient for the reliable assessment of five-year survival outcomes, late-term toxicities, local control, and recurrence rates. Therefore, we did not emphasize these endpoints to avoid potential bias. Furthermore, for patients whose hormone receptor status was initially unknown, pathology specimens were recommended for review in reference pathology centers. However, these patients declined further pathological evaluation; therefore, this missing data could not be resolved. Future prospective randomized trials are needed to better define the optimal boost strategies within hypofractionation regimens and identify patient subgroups most likely to benefit. Incorporation of molecular risk stratification and patient-reported outcomes will be crucial to tailor treatment approaches and balance oncologic benefits with cosmetic outcomes and patient satisfaction. Furthermore, advanced planning techniques such as SIB-IMRT may help minimize boost-associated toxicities while preserving excellent local control and cosmetic outcomes [29,30]. These considerations will be key to refining DCIS treatment paradigms in the future to ensure both efficacy and a patient-centered approach.

## Conclusions

In this 12-year, single-center experience, both hypofractionated and conventional radiotherapy regimens demonstrated comparable local control and low rates of late toxicity in patients with DCIS. While boost administration improved local control in high-risk subgroups, it also increased acute toxicity in patients receiving HFRT. These findings underscore the need for individualized treatment approaches that balance oncologic benefits with potential toxicity risks. Future prospective trials and molecular profiling may further optimize patient selection and refine radiotherapy protocols to ensure the best possible outcomes for DCIS patients.
